# Comparison of Surface Functionalization of PLGA Composite to Immobilize Extracellular Vesicles

**DOI:** 10.3390/polym13213643

**Published:** 2021-10-22

**Authors:** Jiwon Woo, Kyoung-Won Ko, Seung-Gyu Cha, Yun Heo, Dong Keun Han

**Affiliations:** Department of Biomedical Science, CHA University, 335 Pangyo-ro, Bundang-gu, Seongnam-si 13488, Korea; wldnjs6888@naver.com (J.W.); glintk03@gmail.com (K.-W.K.); chkh8047@naver.com (S.-G.C.); yun.heo@icloud.com (Y.H.)

**Keywords:** biodegradable implant, surface modification, fibronectin, polyethylenimine, polydopamine, immobilization of EVs, endothelial cell

## Abstract

Endothelialization by materials provides a promising approach for the rapid re-endothelialization of a cardiovascular implantation. Although previous studies have focused on improving endothelialization through the immobilization of bioactive molecules onto the surface of biodegradable implants, comparative studies of effective surface modification have not yet been reported. Here, we conducted a comparative study on the surface modification of poly(lactide-co-glycolide) (PLGA)-based composites to graft mesenchymal stem cell-derived extracellular vesicles (MSC-EVs) using three different materials, fibronectin (FN), polyethylenimine (PEI), and polydopamine (PDA), which have different bond strengths of ligand–receptor interaction, ionic bond, and covalent bond, respectively. Further in vitro analysis exhibited that MSC-EVs released from all modified films sustainably, but the MSC-EVs grafted onto the surface coated with PEI are more effective than other groups in increasing angiogenesis and reducing the inflammatory responses in endothelial cells. Therefore, the overall results demonstrated that PEI is a desirable coating reagent for the immobilization of MSC-EVs on the surface of biodegradable implants.

## 1. Introduction

Cardiovascular disease is currently identified as a major cause of morbidity and mortality worldwide, and this situation is expected to continue for years to come, placing a significant strain on global health resources [1]. It is well known that poor diet, smoking, obesity, and lack of physical activity are various modifiable risk factors for cardiovascular disease, all of which lead to pro-inflammatory conditions [2,3]. In fact, previous studies have established a crucial role of the inflammatory response in the pathogenesis of cardiovascular diseases through inducing endothelial cell dysfunction [4,5,6].

The vascular endothelium is a monolayer of endothelial cells (ECs) that surrounds the entire luminal surface of the blood vessels and forms the regulatory interface between the circulating blood components and the underlying tissue compartments. Under normal conditions, the endothelium retains a non-adhesive and non-thrombotic surface on which blood cells slide with minimal interaction with the ECs. During inflammation, ECs are activated in response to pro-inflammatory stimuli that promote a robust increase in the expression level of cell adhesion molecules (CAMs) [7,8]. Circulating leukocytes are attached to the inflamed endothelium, where they firmly interact through various classes of CAMs. The initial rolling process of leukocytes is mediated mainly by the selectins expressed on activated ECs and P-selectin glycoprotein ligand-1 (PSGL-1) on the leukocyte surface, whereas firmer adhesion is mediated largely by vascular cell adhesion molecule-1 (VCAM-1) and intercellular adhesion molecule-1 (ICAM-1) on the ECs [9,10,11]. Therefore, the inhibition of adhesion and recruitment of leukocytes to ECs could have a beneficial effect on inflammatory vascular diseases.

Mesenchymal stem cell (MSC)-based approaches have been established as a potential therapy in regenerative medicine [12,13,14,15]. MSCs are multipotent stem cells that are obtained from various tissues, such as adipose tissue, bone marrow, peripheral blood, placenta, and umbilical cord, and can be differentiated into a variety of cell types. Initially, the therapeutic effect of MSCs was attributed to their capability to engraft in damaged tissues and differentiate into functional cells. However, accumulating evidence has revealed that biological effects observed in MSCs treatment are likely due to their secreted factors [16,17,18]. In particular, extracellular vesicles (EVs) derived from MSCs have emerged as key mediators for the therapeutic effects. EVs are heterogeneous vesicles enclosed in phospholipid bilayer that play a key role in cell-to-cell communication by carrying DNA, non-coding RNAs, proteins, and lipids out of cells [19,20]. As a potential alternative for regenerative medicine, EVs have the theoretical advantage of being a safer regenerative tool when compared to cell-based therapies. Recently, surface modification with nanovesicles such as EVs and liposomes is reported to improve the property of implantable medical devices [21,22,23,24,25]. However, one of the major challenges of the application of EVs in medical devices is that free EVs do not allow durable retention at damaged sites, because it is hard to modulate burst release within several hours post-implantation and achieve release in a sustained manner.

**Scheme 1:**
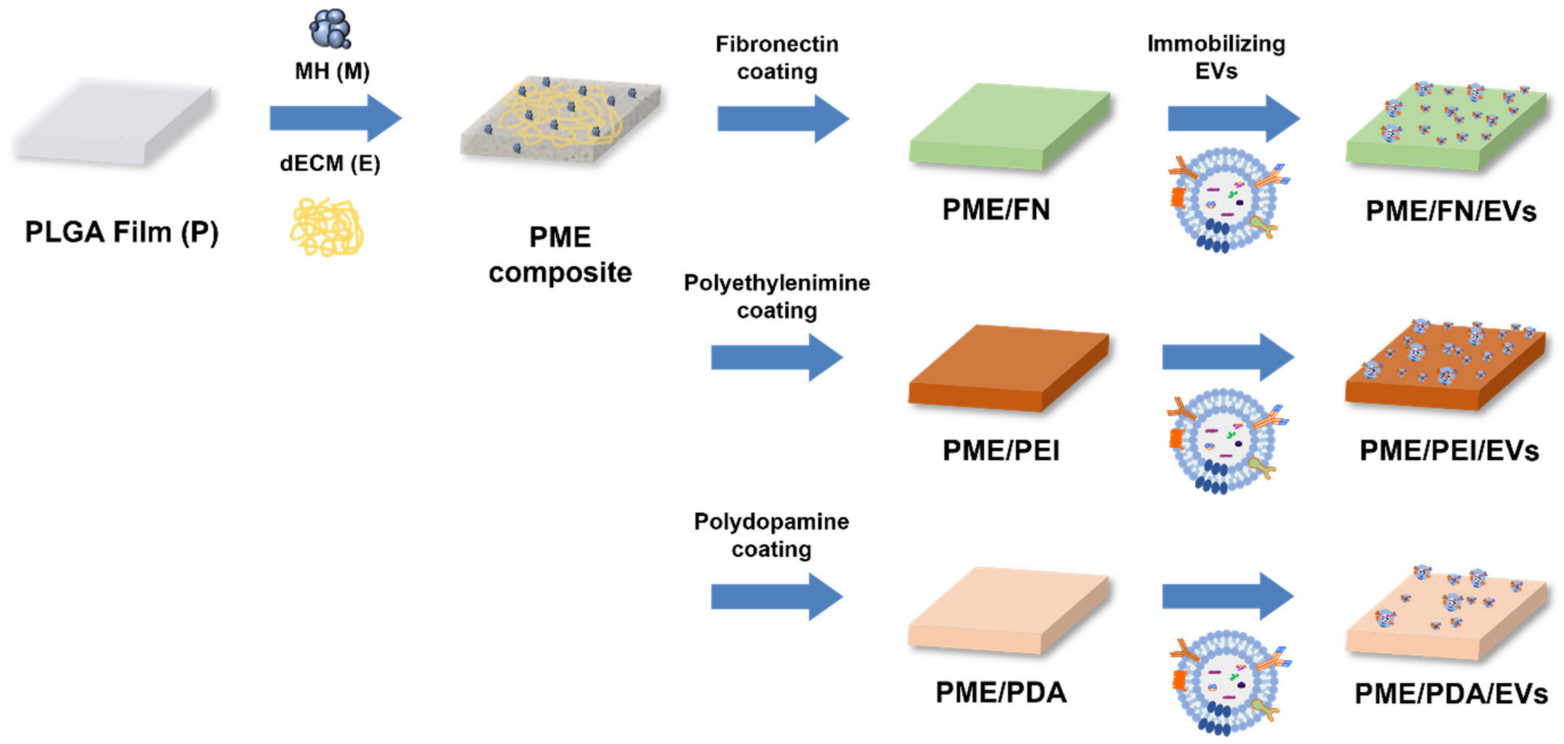
Comparative analysis of the different surface modification for immobilization of extracellular vesicles (Fibronectin for ligand–receptor interaction; Polyethylenimine for ionic bonding; Polydopamine for covalent bonding).

In this study, we investigated the effects of differential affinity immobilization of MSC-derived EVs on poly(lactide-*co*-glycolide) (PLGA) composite films using surface coating with fibronectin (FN), polyethylenimine (PEI), and polydopamine (PDA) on the restoration of endothelial function (Scheme 1). FN is an abundant ECM component containing three kinds of repeated modules, arginine–glycine–aspartic acid (RGD), and several binding sites that facilitate interactions with diverse extracellular components including integrins [26,27]. PEI is a cationic polymer that contains repeating units composed of an amine group and can be strongly interacted with anionic components [28]. In addition, PDA contains ligands consisting of phenyl, catechol, and amine groups, and these ligands can be strongly bound to various substrates by hydrophobic interaction, hydrogen bonding, and electrostatic interaction [29]. The purpose of this study is, therefore, to evaluate that the different strength of the interaction of MSC-derived EVs correlates with the restoration of endothelial function.

## 2. Materials and Methods

### 2.1. Materials

Poly(d,d-lactide-*co*-glycolide) (PLGA, LA/GA = 75:25, MW 150,000) was obtained from Evonik Ind. (Essen, Germany). Magnesium hydroxide (MH), branched polyethylenimine (PEI), and polydopamine (PDA) were purchased from Sigma-Aldrich (St. Louis, MO, USA). Decellularized kidney extracellular matrix (dECM) was kindly provided by Prof. Tae Gyun Kwon from Kyungpook National University Hospital, Korea. Human fibronectin native protein (FN) was obtained from Gibco (Waltham, MA, USA). ELISA kits (R&D Systems, Minneapolis, MN, USA) were used to analyze the cytokine concentrations according to the manufacturer’s instructions.

### 2.2. Cell Culture

Human coronary artery endothelial cells (HCAECs) were purchased from Lonza (Walkersville, MD, USA) and cultured in endothelial growth medium-2 (EGM-2 MV, Lonza). Human umbilical cord-derived MSCs were provided by CHA Biotech, Co. Ltd. (Seongnam, Korea). Cells were cultured in DMEM with low glucose supplemented with 10% fetal bovine serum (FBS, Hyclone, Logan, UT, USA) and 1% antibiotic-antimycotic solution (AA, Gibco, Amarillo, TX, USA). Cells were cultivated at 37 °C in a humidified atmosphere containing 5% CO_2_.

### 2.3. Fabrication of PLGA Composite Films

The PLGA composite films containing MH and dECM were fabricated using solvent casting. Briefly, PLGA was dissolved in chloroform at 10 wt% of PLGA, and MH and dECM were added at 20 and 10 wt% of the PLGA mass, respectively. The dECM powder was prepared by the decellularization of the porcine kidney as previously described [30]. To prepare the coating solution, FN (50 μg/mL) and PEI (200 μg/mL) were diluted with PBS solution, and PDA (1 mg/mL) was dissolved in a Tris buffer (pH 8.5). Before the coating, for hydrolysis, the solution was soaked in 70% ethanol for 10 min and washed twice with distilled water, then immersed in MES buffer for 2 h. The PLGA composite films were immersed in each solution at room temperature overnight, washed twice with PBS solution and then allowed to dry at room temperature. For the immobilization of MSC-EVs, 200 μg EVs were loaded onto the hydrated composite film.

### 2.4. Characterization of PLGA Composite Films

The surface of the composite films was analyzed using attenuated total reflectance-Fourier transform infrared (ATR-FTIR; SIGMA, Carl Zeiss, Oberkochen, Germany) and X-ray photoelectron spectroscopy (XPS, Nexsa, ThermoFisher Scientific, Waltham, MA, USA). The chemical bonding of the surface was determined by ATR-FTIR in the range of 650–4000 cm^−1^ and scan speed (0.2 cm/s). For XPS condition, microfocus X-ray (1486.6 eV) was used with charging correction (adventitious carbon, 284.8 eV) and dual neutralizer (Ar ion + electrons) [31,32]. The pH changes were measured by a pH meter (Mettler Toledo, Columbus, OH, USA) in 5 mL of PBS solution containing Proteinase K at 37 °C for 14 days. The hydrophilicity of the surface PME composite film was examined by a contact angle analyzer (Phoenix 300, Surface Electro Optics, Suwon, Korea). Micro-BCA (Pierce, Rockford, IL, USA) was used for each quantitative analysis coated on the film. For scanning electron microscopy (SEM), EV-immobilized films were fixed with 2.5% glutaraldehyde in PBS solution for 2 h and then imaged using field emission-scanning electron microscopy (FE-SEM; SIGMA, Carl Zeiss) at an acceleration voltage 5 kV. Iridium was used as the coating material, Leica EM ACE600 as the coating technique, and 6 nm as the thickness of the conductive coating. Released EVs were quantified using a Micro-BCA (Pierce).

### 2.5. Isolation and Characterization of MSC-Derived EVs

The culture medium of UC-MSCs was changed to exosome-depleted medium (i.e., FBS was centrifuged at 100,000× *g* for 18 h), and cultures were incubated for 48 h. The conditioned medium was collected from each dish, and 20 mL of fresh medium was added to each dish for another 12 h of culture. Cell debris and larger vesicles were pelleted at 1000 g for 30 min, the supernatant was filtered through a 0.22 μm PES membrane filter, and then the conditioned medium was subjected to a tangential flow filtration (TFF) system with a 500 kDa hollow fiber membrane (KR2i TFF system, Repligen, Waltham, MA, USA). Isolated EVs were concentrated with Amicon Ultra-2 10 K (Merck Millipore, Billerica, MA, USA). For transmission electron microscopy (TEM) analysis, EVs were loaded on a carbon-coated copper grid (Electron Microscopy Sciences, Washington, PA, USA). Samples were incubated with 2% uranyl acetate solution and washed twice with distilled water. EV images were recorded using an FEI Tecnai Spirit G2 (FEI Company, Hillsboro, OR, USA) at an acceleration voltage of 80 kV, and the size distribution and particle concentration were analyzed by nanoparticle tracking analysis (NTA) using a Zetaview (Particle Metrix GmbH, Meerbusch, Germany). Single particle interferometric imaging measurement was performed using the ExoView platform (Nanoview Biosciences, Boston, MA, USA). Data are representative of three independent experiments.

### 2.6. Western Blot Analysis

Samples were mixed with a Laemmli buffer, heated at 105 °C for 10 min, and loaded onto a 10% sodium dodecyl sulfate-polyacrylamide gel electrophoresis (SDS-PAGE) and transferred to NC membranes. After blocking, sequential incubation was carried out with primary antibodies, and horseradish peroxidase (HRP)-linked secondary antibodies, and then blots were developed using an enhanced chemiluminescence solution for 5 min, after which they were scanned with a ChemiDoc XRS system (Bio-Rad, Hercules, CA, USA). The experiment was performed in triplicate for three samples of each group. The following antibodies were used: CD63 (Abcam, Cambridge, MA, USA), CD81 (Santa Cruz Biotechnology, Inc., Santa Cruz, CA, USA), and CD9 (Santa Cruz Biotechnology, Inc., Santa Cruz, CA, USA) were used as primary antibodies, and HRP-conjugated anti-rabbit or anti-mouse antibodies (Cell Signaling Technology, Danvers, MA, USA) were used as secondary antibodies.

### 2.7. Wound Healing Assay

HCAECs were seeded into a 12-well plate at a density of 2 × 10^5^ cells per well. When the cells reached 100% confluence, the monolayers were scratched using a sterile 1000 μL pipet tip. After disruption, monolayers were gently washed twice with PBS solution to remove cell debris. Subsequently, the cells were treated with EVs (200 μg/mL). The plates were incubated at 37 °C in a 5% CO_2_ air atmosphere for 16 h. Three samples of each group were tested in duplicate. The migration area of cells was measured by using ImageJ.

### 2.8. Leukocyte Adhesion Assay

HCAEC monolayers, grown as described earlier, were established in culture plates. Lipopolysaccharide (LPS) and the composite films were added. After being incubated for 24 h, the monolayers were incubated with 2 × 10^5^ THP-1 cells labeled with Calcein-AM (Invitrogen, Carlsbad, CA, USA) for 1 h. After incubation, non-adherent cells were removed by washing with PBS solution twice. Three samples of each group were tested in duplicate. A total of three random regions were photographed, and the numbers of adhered cells were directly counted.

### 2.9. Tube Formation Assay

Matrigel basement membrane matrix (BD Biosciences, San Jose, CA, USA) was added to a 24-well plate and solidified at 37 °C for 1 h. Then, 1×10^5^ HCAECs per well were seeded and cultured with the composite films. After incubating at 37 °C and 5% CO_2_ for 16 h, the cells were stained with Calcein AM (Invitrogen, Waltham, MA, USA). Tube formation assay was performed in triplicate for each group. The number of branch points was measured using the angiogenesis plug-in of ImageJ.

### 2.10. RNA Extraction and Quantitative Real-Time PCR

Total cellular RNA was isolated using an AccuPrep Universal RNA Extraction Kit (Bioneer, Daejeon, Korea). The 1 μg of isolated RNA was reverse-transcribed into cDNA using a PrimeScript RT reagent kit (perfect real-time) (TaKaRa Biotechnology, Kusatsu, Japan), and quantitative real-time PCR was performed using a Power SYBR green PCR master mix (Applied Biosystems, Carlsbad, CA, USA) on a QuantStudio3 real-time PCR system (Applied Biosystems, Waltham, MA, USA). Ct values were normalized against the 18s rRNA and calculated using a 2^−ΔΔCt^ method. The description of the primer pairs used in this study was given in Table S1.

### 2.11. Statistical Analysis

Quantitative data are expressed as the mean ± standard deviation (SD). Differences between more than three groups were compared using one-way ANOVA and Tukey post hoc tests using GraphPad Prism 5.0 (GraphPad Software, Inc., San Diego, CA, USA). *p* < 0.05 was considered statistically significant.

## 3. Results and Discussion

### 3.1. Characterization of PLGA Composite Films

**Figure 1:**
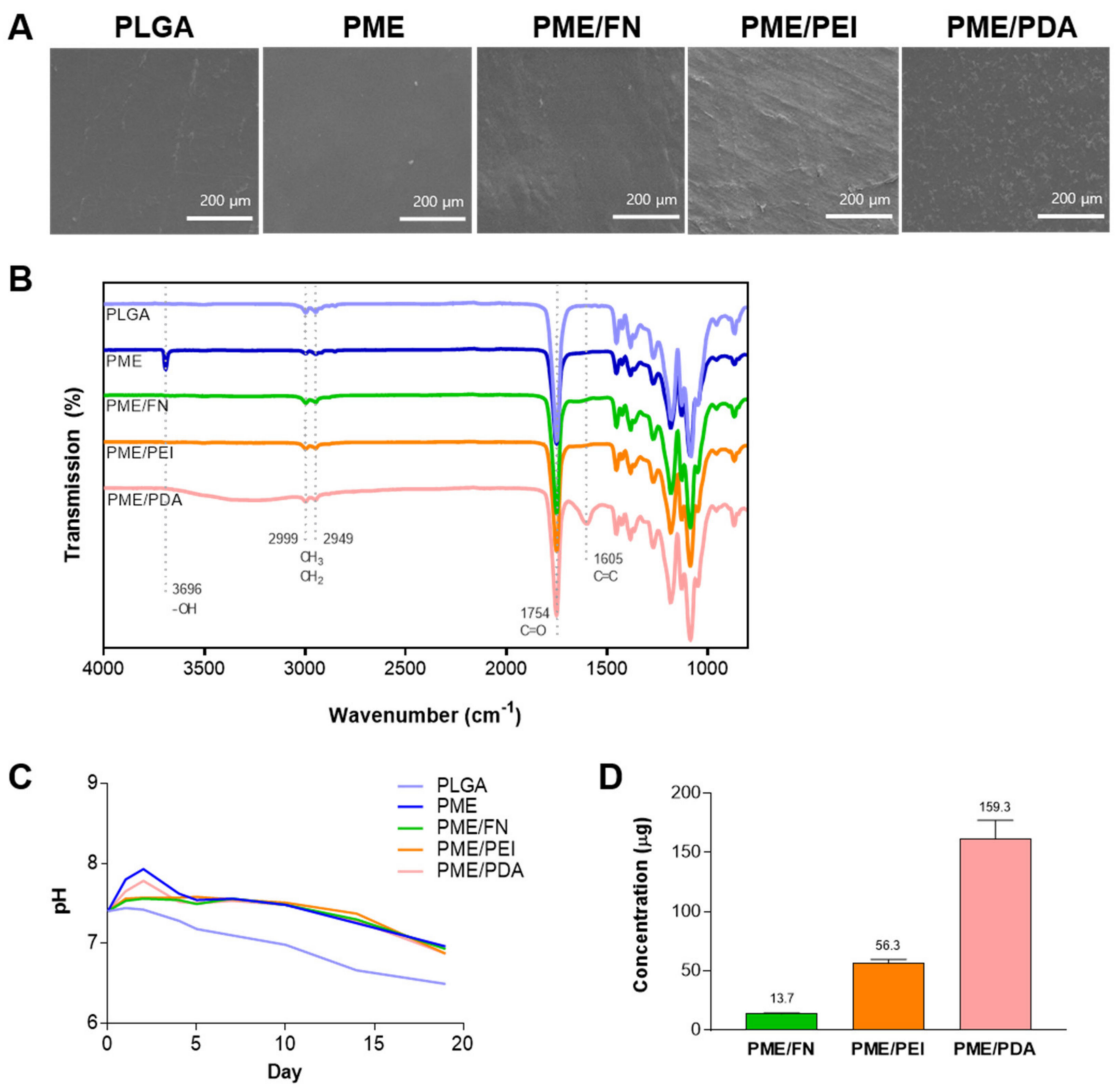
Characterization of surface-modified composite films. (**A**) Representative SEM images of the PLGA composite films. (**B**) ATR-FTIR spectra. (**C**) The changes in pH. (**D**) The total amount of coating materials on the surface of composites. Scale bars = 200 μm. The data are expressed as mean ± SD (*n* = 3).

PLGA is widely recognized as a biocompatible and biodegradable biomaterial and has already been widely used in clinics [29]. We previously established that PLGA-based biodegradable implants containing magnesium hydroxide (MH) effectively alleviated the adverse effects induced by acidic PLGA byproducts [30,31]. In addition, decellularized extracellular matrix (dECM) is extensively used as a platform for the bioengineering of medical implants to enhance and control the interaction between implants and host tissues [32]. Therefore, PLGA/MH/dECM composite film was fabricated by solvent casting, and then the surface of composite films with fibronectin (FN), polyethylenimine (PEI), and polydopamine (PDA) was coated as described in Section 2. The surface-modified composite films were characterized using XPS, ATR-FTIR, SEM, and water contact angle (WCA) measurements. As shown in Table 1, PLGA showed the elemental composition of carbon (73.46%) and oxygen (25.18%). The composition of PME film was similar to PLGA film. After surface modification with FN, PEI, and PDA, a new composition was observed which represented nitrogen molecules. The molecules of FN and PEI displayed a higher nitrogen composition than PDA due to the higher nitrogen content in the molecular structure. In comparison to PLGA film (89.3 ± 1.0°), the wettability of PME, PME/FN, PME/PEI, and PME/PDA significantly decreased with WCA of 73.7 ± 1.0, 58.81 ± 1.0, 69.5 ± 4.2, and 47.5 ± 1.3°, respectively. According to the SEM images, surface roughness changes were induced during the process of the film coating (Figure 1A). The neutralization effect of PLGA/MH/dECM composite films was confirmed with the maintenance of neutral pH, contrary to a PLGA film with low pH (Figure 1B). The surface composition of modified composite films was analyzed using ATR-FTIR. The spectra of modified composite films were similar to that of PLGA, but the PME spectrum contained the O-H stretching vibration around 3700 cm^−1^ because of O-H stretching in magnesium hydroxide. After PDA coating, a new broad peak was observed from 3000 to 3500 cm^−1^, which represents hydroxyl groups (Figure 1C). In addition, the quantification of the number of coating substrates on the composite films was performed with a micro-BCA assay (Figure 1D). These results indicated the successful incorporation of coating substrates on the PLGA/MH/dECM composite films.

### 3.2. Characterization of MSC-Derived EVs

**Figure 2:**
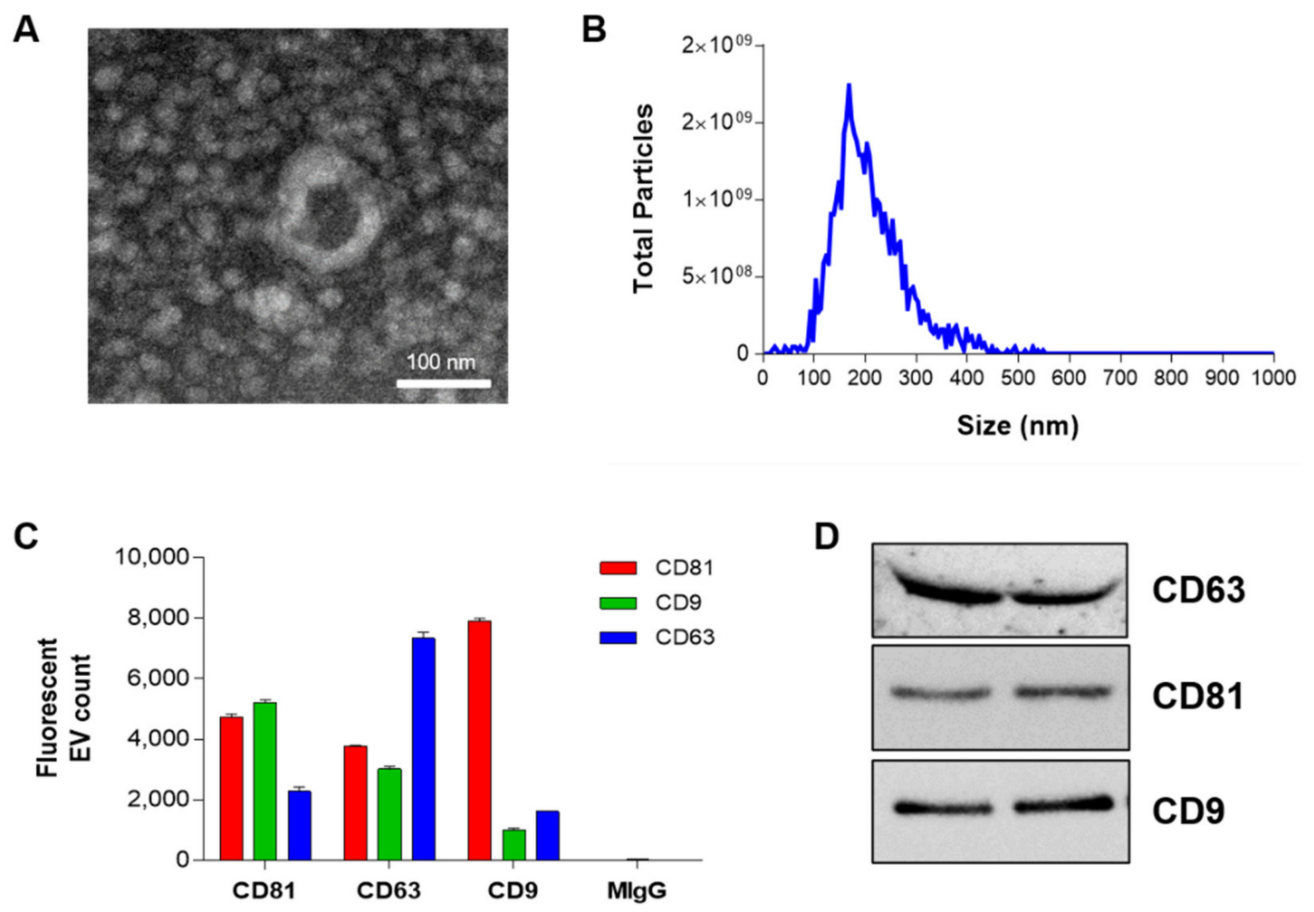
Characterization of EVs. (**A**) Evaluation of morphology by TEM. Scale bars = 100 nm. (**B**) Averaged NTA-determined size distributions. (**C**) ExoView shows the expression of CD9, CD63, and CD81 in MSC-derived EVs. (**D**) Western blot analysis of the exosomal surface markers.

EVs from the conditioned medium of umbilical cord-derived MSCs (UC-MSCs) were isolated using the TFF system. The resulting EVs were round in shape with similar uniform size and vesicular-like shape (Figure 2A). NTA analysis revealed similar hydrodynamic sizes, which averaged 167.5 nm (Figure 2B). To confirm the presence of exosomal proteins and specific surface markers, EVs were analyzed with ExoView and Western blot analysis. Using the ExoView affinity microarray platform, EVs mainly interacted with antibodies against each tetraspanin, and then fluorescently labeled by secondary antibodies for the three tetraspanins, CD63, CD81, and CD9 (Figure 2C). The results implied that isolated EVs were all positive for the typical exosome markers. Additionally, the expression of the exosome markers CD63, CD81, and CD9 was confirmed by Western blot analysis (Figure 2D).

### 3.3. Immobilization of EVs on the Surface-Modified Composite Films

Local administration of MSC-EVs can preserve high concentrations at damaged sites. Nevertheless, repeated treatment requests for maintaining the effective concentration to improve tissue regeneration [33]. Several studies have demonstrated that scaffold-anchored EVs accelerated the restoration of damaged tissues through sustained release and enhanced bioactivities [25,27]. However, the effect of differences in the relative binding affinity of EVs to the scaffold that could control the release profile of anchored EVs is still unknown.

**Figure 3:**
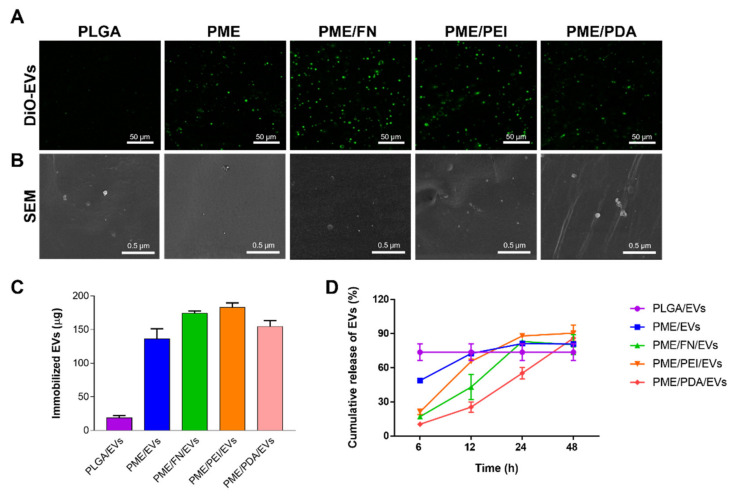
Immobilization of EVs onto the composite. (**A**) Representative confocal images showing the distribution of DiO-labeled EVs on the surface of composite films. Scale bars = 50 μm. (**B**) Representative SEM images of EV-grafted films. Scale bars = 0.5 μm. (**C**) Quantification of immobilized EVs on the composite films. (**D**) EVs release profile.

To induce a difference in bonding strength between EVs and the film surface, three different types of interactions, ligand–receptor interaction (FN-coated film and EVs), ionic bonding (PEI-coated film and EVs), and covalent bonding (PDA-coated film and EVs), were introduced to modify the surface of the composite films. After the coating processes, equal amounts of DiO-labeled EVs were loaded on the surface-modified composite films. The SEM images and laser confocal scanning microscopic images demonstrated that the EVs were homogeneously distributed on the surface of the PME composite film and surface-modified PME films after the immobilization process, whereas only a few EVs were observed in the PLGA film (Figure 3A,B). However, an unexpected difference in the amount of bound EVs was observed between the groups. The total amounts of grafted EVs onto the PLGA, PME, PME/FN, PME/PEI, and PME/PDA films were 18.5, 136.2, 174.1, 183.1, and 154.6 μg, respectively (Figure 3C). The grafted EVs exhibited burst release from the PLGA film within 6 h. In the PME group, 48% of bound EVs were released within 6 h, and almost all EVs were depleted within 24 h. The PME composite film contains decellularized ECM that can interact with various other components including EVs, resulting in a relatively higher amount of EVs, and was grafted onto the PME composite film compared to PLGA film, as previously reported [28]. In contrast, EVs grafted onto the surface-modified composite films were released slowly over 48 h, especially the PME/PDA film (Figure 3D). These findings indicated that the surface-modified composite using FN, PEI, and PDA is an efficient carrier for the sustained release of loaded EVs, which is promising for the application in EV-functionalized medical devices.

### 3.4. Effect of Released EVs from Composite Films on EC Function

**Figure 4:**
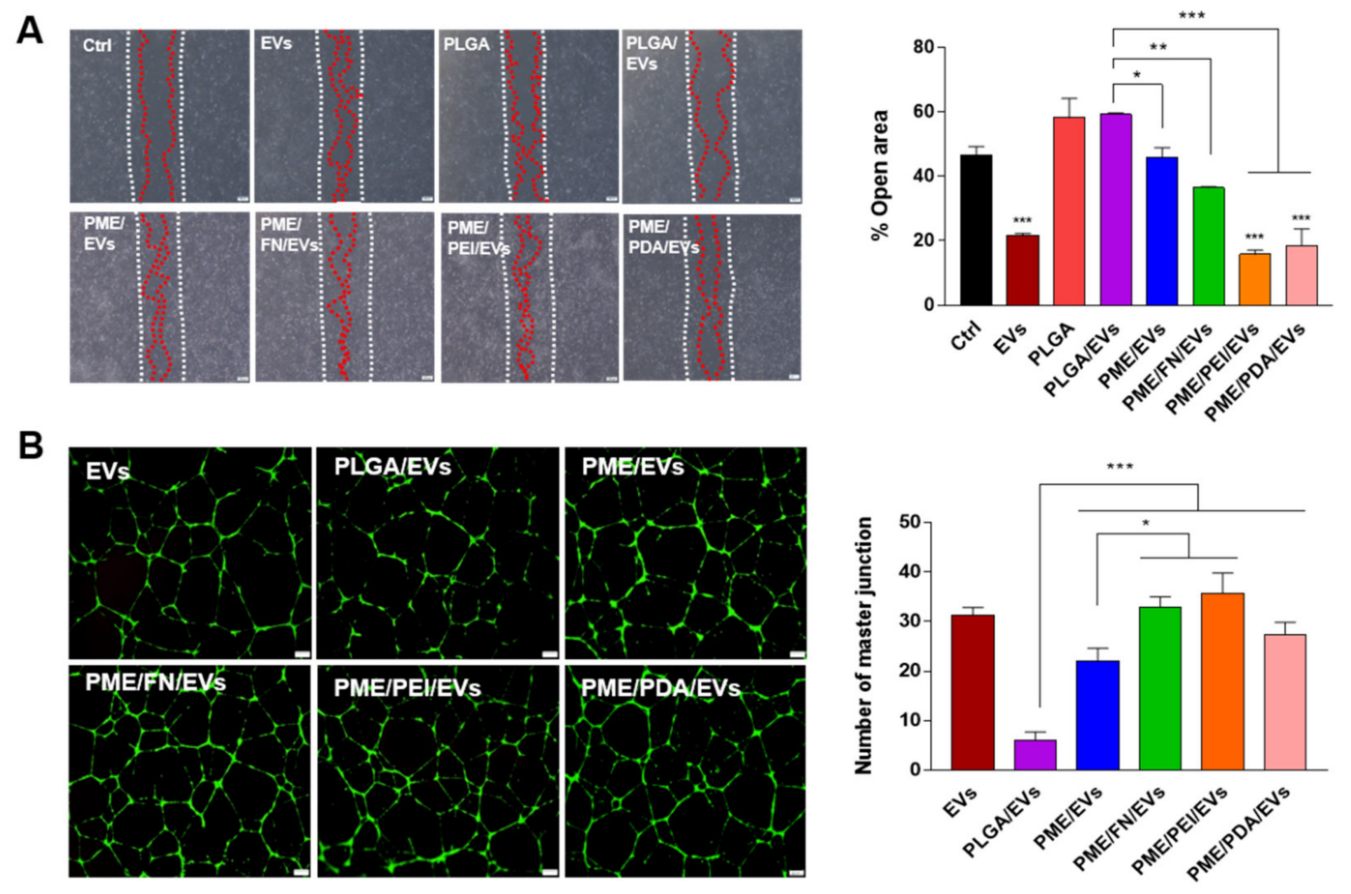
Effect of EVs released from the composite films on angiogenesis. (**A**) Representative images and quantitative analysis of wound healing assay. (**B**) Representative images and quantitative analysis of tube formation assay characterizing number of master junction. Scale bars = 200 μm. The data are expressed as mean ± SD (*n* = 3). * *p* < 0.05; ** *p* < 0.01; *** *p* < 0.001.

Therapeutic angiogenesis is one promising strategy for the treatment of ischemic heart disease, which is the leading cause of death globally [34,35]. To determine the functional impact of EVs released from surface-modified films on angiogenesis, wound healing and tube formation assays were performed in human coronary artery endothelial cells (HCAECs). As shown in Figure 4A, EVs significantly accelerated wound healing compared to the untreated group. Cells incubated with PME/EVs, PME/FN/EVs, PME/PEI/EVs, and PME/PDA/EVs exhibited a significant reduction in the wound area, compared to PLGA/EVs. Notably, the group of PME/PEI/EVs displayed the smallest wound area among the EV-grafted films, thus showing the highest wound closure rate in this experiment. In parallel, tube formation assay indicated that EVs effectively enhanced tube formation of HCAECs, and the cells incubated with PME/FN/EVs, PME/PEI/EVs, and PME/PDA/EVs produced capillary-like tubes compared to PLGA/EVs. Among the surface-modified groups, PME/PEI/EVs showed higher capillary tube formation activity. The differences in angiogenic properties between these groups might be due to the total amount of initial immobilized EVs and release rate. Overall, these results revealed that PEI is the most valuable coating material to immobilize EVs onto the surface of PLGA-based composite among these materials.

### 3.5. Effect of EVs Released from Composite Films on Inflammation

Inflammation was firmly established as a crucial process to the development and complications of cardiovascular diseases. The inflammatory response in endothelial cells involves the recruitment and adhesion of circulating leukocytes to the damaged area, which is regulated by the expression of adhesion molecules [36].

**Figure 5:**
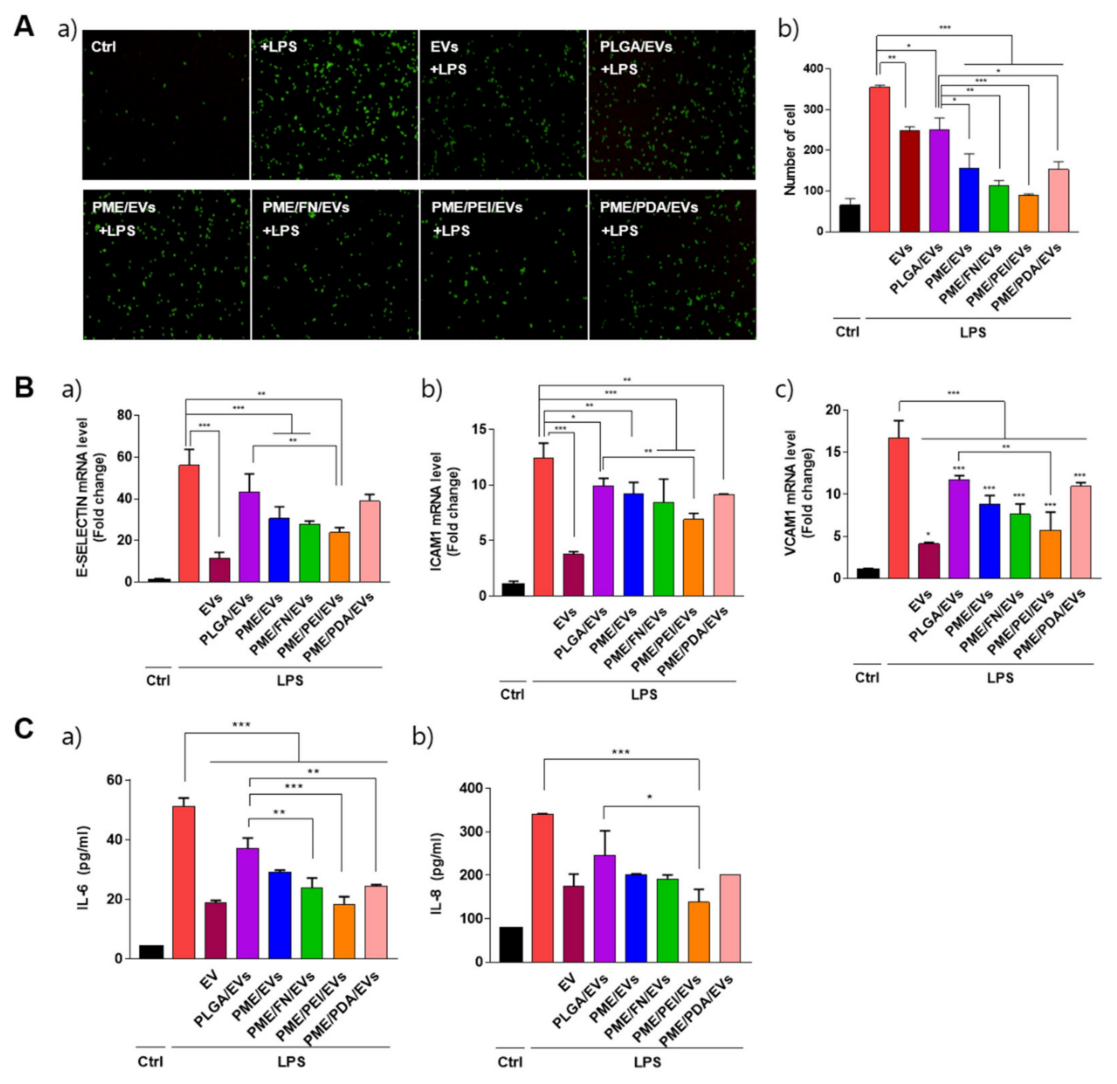
Effect of EVs released from the composite films on inflammation. (**A**) (**a**) Representative images, and (**b**) quantification of THP-1 cells adherent to LPS-activated HCAECs. (**B**) Gene expression levels of cell adhesion molecules. (**a**) E-SELECTIN, (**b**) ICAM1, and (**c**) VCAM1 mRNA expression levels. (**C**) Quantification of secreted pro-inflammatory cytokines. (**a**) IL-6, and (**b**) IL-8 concentrations in cultured medium. The data are expressed as mean ± SD (*n* = 3). * *p* < 0.05; ** *p* < 0.01; *** *p* < 0.001.

To explore the effect of grafted EVs onto the surface-modified films on endothelial activation induced by LPS, the adhesion ability of THP-1 monocytes to LPS-stimulated HCAECs was evaluated. As shown in Figure 5A, the adhesion of THP-1 cells was significantly diminished by EVs. The EVs released from the PME, PME/FN, PME/PEI, and PME/PDA composite films also reduced the adhesion of THP-1 cells, particularly at the composite film coated with PEI. Since the induction of the expression of cell adhesion molecules on the surface of endothelial cells is necessary for the interaction of endothelial cells and leukocytes, the expression levels of cell adhesion molecules, E-SELECTIN, ICAM-1, and VCAM-1 were then measured.

Consistent with leukocyte adhesion assay, EVs significantly decreased the expressions of cell adhesion molecules induced by LPS. Moreover, EVs released from the composite films, PME, PME/FN, PME/PEI, and PME/PDA also reduced expression levels of cell adhesion molecules (Figure 5B). In particular, PME/PEI/EVs displayed a statistically significant reduction in adhesion molecules compared to PLGA/EVs. In addition, LPS-induced expression of pro-inflammatory cytokines, IL-6, and IL8 strikingly decreased in EVs containing groups. Among them, PME/PEI/EVs displayed the best effects on the secretion of pro-inflammatory cytokines (Figure 5C). These results implied that the immobilization of EVs using PEI coating effectively attenuated the induction of inflammation-related factors in LPS-stimulated endothelial cells.

Diomede et al. have previously demonstrated that improved internalization of PEI-modified EVs into the cells may be caused by the ability of PEI, being a shell capable of binding EVs via ionic bonding, to prefer internalization through proteoglycan binding [37]. Although molecular analysis of EVs grafted onto the PEI coated film is not sufficient to understand how its effect of restoration of endothelial function is improved compared with FN-coated film, which grafted a similar amount of EVs, it is possible that PEI on the surface of the secreted EVs might be augmented the internalization of EVs to recipient cells.

Collectively, these results suggested that surface modification with PEI, a cationic polymer, should be promising for immobilizing EVs onto the PLGA-based implantable medical devices.

However, there still exist some limitations in this study. First, further in vivo studies should be performed to evaluate whether EVs grafted onto the PEI-coated composite can restore the endothelial cells. Second, the mechanism behind the improved effect of released EVs from the PEI-modified film is not clearly defined and needs further exploration on the basis of our current studies.

## 4. Conclusions

In this study, we provide intriguing clues into the surface modification of PLGA-based composite for the immobilization of EVs. To establish the optimal conditions for immobilizing EVs, the composite surface is coated with FN, PEI, and PDA. Our findings propose that PEI, which has an intermediate bond strength such as an ionic bond, is the appropriate material to immobilize EVs onto the surface of the PLGA-based composite. Moreover, immobilized EVs on the PEI-coated composite surface were released sustainably, resulting in the improvement of angiogenesis and alleviation of inflammatory responses in endothelial cells. This concept is expected to be applicable to the functionalization of biodegradable implants using EVs.

## Figures and Tables

**Table 1:** Surface chemical composition and water contact angle of the composite films.

Sample	XPS Atomic Composition (%)			Water Contact Angle (Degree)
	C	O	N	
PLGA	73.46	25.18	-	89.3 ± 1.0
PME	73.59	25.61	-	73.7 ± 1.0
PME/FN	64.87	26.93	7.52	58.8 ± 1.0
PME/PEI	63.64	26.82	8.15	69.5 ± 4.2
PME/PDA	78.58	18.75	2.67	47.5 ± 1.3

## Data Availability

The data that support the findings of this study are available on request from the corresponding author D.K. Han.

## Supplementary Materials

The following are available online at https://www.mdpi.com/article/10.3390/polym13213643/s1, Table S1: List of primers sequences used for quantitative real-time PCR analysis.
