# Supplementary material for: Comparison of Surface Functionalization of PLGA Composite to Immobilize Extracellular Vesicles
Source: Polymers (Basel). 2021 Oct 22;13(21):3643. doi: 10.3390/polym13213643 (PMC8587822; doi:10.3390/polym13213643)
Supplement: Supplementary file 1 [file polymers-13-03643-s001.zip › polymers-1421038-supplementary.pdf]

Supplementary information

Comparison of surface functionalization of PLGA composite to immobilize extracellular vesicles

Jiwon Woo¶, Kyoung-Won Ko¶, Seung-Gyu Cha, Yun Heo, and Dong Keun Han*

Table S1. List of primers sequences used for quantitative real-time PCR analysis

Gene	Sequence (5′-3′)
h 18s Rrna	F: gcaattattccccatgaacg<br>R: gggacttaatcaacgcaagc
h E-SELECTIN	F: accagcccaggttgaatg<br>R: ggttggacaaggctgtgc
h ICAM-1	F: ccttcctcaccgtgtactgg<br>R: agcgtagggtaaggttcttgc
h VCAM-1	F: tgcacagtgacttgtggacata<br>R: gccaccactcatctcgattt

F: Forwad, R: Reverse
